# Intraindividual Variability in Inhibitory Function in Adults with ADHD – An Ex-Gaussian Approach

**DOI:** 10.1371/journal.pone.0112298

**Published:** 2014-12-05

**Authors:** Dennis Gmehlin, Anselm B. M. Fuermaier, Stephan Walther, Rudolf Debelak, Mirjam Rentrop, Celina Westermann, Anuradha Sharma, Lara Tucha, Janneke Koerts, Oliver Tucha, Matthias Weisbrod, Steffen Aschenbrenner

**Affiliations:** 1 Department of Clinical Psychology and Neuropsychology, SRH Klinikum, Karlsbad-Langensteinbach, Germany; 2 Psychiatric Department, SRH Klinikum, Karlsbad-Langensteinbach, Germany; 3 SüdWestAkadamie für Neuropsychologie (SWAN), Heidelberg, Germany; 4 Section of Experimental Psychopathology and Neurophysiology, Department of child and adolescent Psychiatry, University of Heidelberg, Germany; 5 Department of Clinical and Developmental Neuropsychology, University of Groningen, Groningen, The Netherlands; 6 Schuhfried GmbH, Mödling, Austria; University Medical Center Groningen UMCG, Netherlands

## Abstract

**Objective:**

Attention deficit disorder (ADHD) is commonly associated with inhibitory dysfunction contributing to typical behavioral symptoms like impulsivity or hyperactivity. However, some studies analyzing intraindividual variability (IIV) of reaction times in children with ADHD (cADHD) question a predominance of inhibitory deficits. IIV is a measure of the stability of information processing and provides evidence that longer reaction times (RT) in inhibitory tasks in cADHD are due to only a few prolonged responses which may indicate deficits in sustained attention rather than inhibitory dysfunction. We wanted to find out, whether a slowing in inhibitory functioning in adults with ADHD (aADHD) is due to isolated slow responses.

**Methods:**

Computing classical RT measures (mean RT, SD), ex-Gaussian parameters of IIV (which allow a better separation of reaction time (mu), variability (sigma) and abnormally slow responses (tau) than classical measures) as well as errors of omission and commission, we examined response inhibition in a well-established GoNogo task in a sample of aADHD subjects without medication and healthy controls matched for age, gender and education.

**Results:**

We did not find higher numbers of commission errors in aADHD, while the number of omissions was significantly increased compared with controls. In contrast to increased mean RT, the distributional parameter mu did not document a significant slowing in aADHD. However, subjects with aADHD were characterized by increased IIV throughout the entire RT distribution as indicated by the parameters sigma and tau as well as the SD of reaction time. Moreover, we found a significant correlation between tau and the number of omission errors.

**Conclusions:**

Our findings question a primacy of inhibitory deficits in aADHD and provide evidence for attentional dysfunction. The present findings may have theoretical implications for etiological models of ADHD as well as more practical implications for neuropsychological testing in aADHD.

## Introduction

Attention-deficit/hyperactivity disorder (ADHD) begins in childhood and is characterized by developmentally inappropriate and pervasive behavioral symptoms of hyperactivity, impulsivity and inattention [1]. The disorder, with an estimated prevalence of 3–5% in childhood, goes along with functional impairments across multiple academic as well as social domains, resulting in a large burden for individuals, families and society [2].

About 50% of children diagnosed with ADHD (cADHD) show a partial remission of symptoms but still suffer from persisting behavioral and emotional problems. In at least 15% of cADHD the disorder persists into adulthood. Similar to children and adolescents, symptoms in adult ADHD (aADHD) profoundly impair functioning in not only social and academic, but also occupational areas [3].

### 1. ADHD and inhibition

There are few comprehensive theories of ADHD, which explain and allow predictions about neuropsychological functioning. One of the most prominent models was proposed by Barkley in 1997. It focuses on deficient inhibitory control as the core deficit, secondarily disrupting other executive processes, and ultimately resulting in typical behavioral symptoms of ADHD [4]. Neuropsychological support for this hypothesis has been mainly derived from computerized inhibitory tests like the Stop Signal [5] or the GoNogo task [6].

The GoNogo task basically requires continuous speeded discrimination between two stimuli and the decision to respond (Go-Stimulus) or to withhold the response (Nogo-Stimulus). By increasing the frequency of Go-Trials at the expense of Nogo trials, GoNogo tasks create a prepotency towards responding. Consequently, selective-attention demands are reduced, while the subjects have to sustain attention and to actively withhold or inhibit a motor response in case of the less frequent Nogo-Stimuli. If subjects respond to trials where they are required to withhold the response they commit commission errors, which are taken as an index of failed inhibitory control. In contrast, omission errors occur when subjects do not respond to trials where they are required to respond and normally indicate deficits in sustained attention [7]. Using the GoNogo Task, Wodka, Mahone, Blankner, Larson, Fotedar, et al. [6] found higher rates of commission errors in cADHD compared to control children and therefore suggest response inhibition to be a primary deficit in cADHD. However, the majority of studies which applied GoNogo tasks in aADHD did not find more commission errors compared to normal controls. In fact, Epstein, Casey, Tonev, Davidson, Reiss, et al. [8] revealed a trend for more omission errors in aADHD. Though discussed lively, this is basically in line with longitudinal studies suggesting a decline of impulsivity and hyperactivity with age in ADHD while symptoms of inattention are rather stable across the life span [9].

Longer reaction times in GoNogo tasks however are more difficult to interpret: a pattern of slower performance – in combination with non-significant increases in the number of commission errors – may indicate some sort of compensatory process, helping to avoid elementary inhibitory dysfunction [7]. However, another explanation for an overt slowing in inhibitory tasks may be an increased intraindividual variability (IIV) in inhibitory function in ADHD [10]–[13]. Though being a contentious issue, increased IIV was linked to some overly slow responses largely influencing mean RT and suggested to mirror deficits in sustained attention. Such increased variability of information processing in ADHD was also found in other computerized tasks like choice discrimination, attention or working memory [14] and is therefore most likely not an implication of inhibitory dysfunction. In the following we will shortly introduce IIV and show how differences in IIV may complicate the interpretation of results on inhibitory function [15].

### 2. Classical measures of intraindividual variability (IIV) and inhibitory function in ADHD

IIV refers to short-term changes of a person's performance on a single task measured on multiple occasions and can be defined as within-person inconsistency that cannot be accounted for by systematic and more enduring changes attributable to development, learning or fatigue [16]. Recent research provides systematic evidence that IIV – as a reliable measure of the stability of information processing – is fundamentally disturbed in ADHD [17].

**Figure 1:**
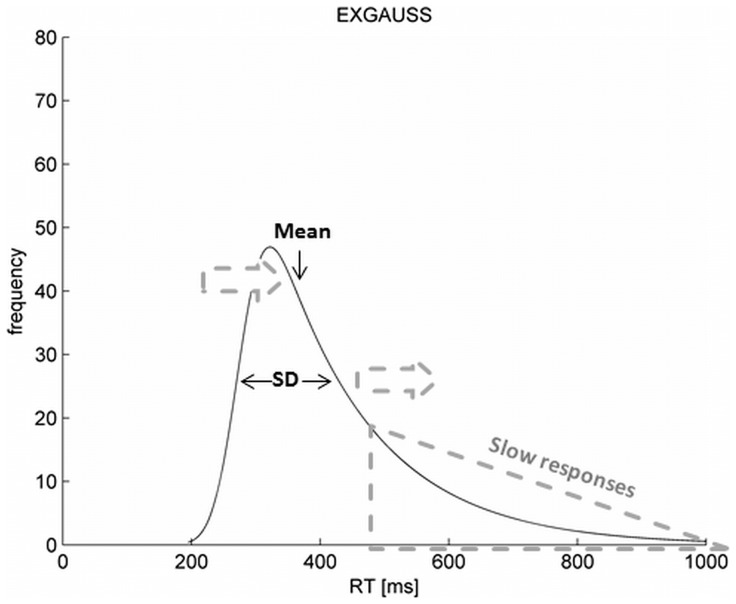
Exemplary distribution of RT and relation of Mean, SD and the number of slow responses. **NOTE**: Dotted grey arrows illustrate a rightward shift of mean RT and an increase of the SD of RT as a consequence of an increasing number of slow responses forming the right tail of the distribution.

Classical neuropsychological measures like the mean reaction time (mRT) and the standard deviation of reaction time (SD) as the most common and easy to compute variability measure are difficult to interpret because they are strongly correlated [18]. Consequently, mRT and SD do not distinguish between speed and variability. Moreover, as reaction times (RT) are not normally distributed, the mRT and SD may miss systematic aspects of the data due to averaging procedures. With regard to ADHD both behavioral and cognitive findings in children suggest that response patterns in patients are characterized by some overly slow responses falling within the right tail of the RT distribution [19] and therefore differ from control subjects. This pattern offers a possible explanation for both longer mean RT and increased SD without assuming a substantial general slowing of responses in ADHD (see Figure 1 below).

Given the reports of non-significant differences in commission errors in aADHD compared to controls, the absence of a general slowing in inhibitory tasks in aADHD would question the assumption of fundamental inhibitory dysfunction. However, more sophisticated measures of IIV are needed to quantify these response patterns and to adequately consider their impact on inhibitory tasks.

### 3. Distributional measures of IIV in ADHD

To circumvent the above-mentioned problems associated with classical measures, more fine grained analyses of the distribution of RT have been used in recent studies examining cADHD. The so called ex-Gaussian distribution is described by the convolution of a normal and an additional exponential function. Fitting the ex-Gaussian function to empirical RT data provides estimates of three independent parameters: (1) Mu (μ) represents the mean of the normal component and mainly reflects average performance. (2) Sigma (σ) corresponds to the SD of the normal component and indicates variability of performance. (3) Tau (τ) corresponds to the variability of the exponential function and reflects extremes in performance [20]. In other words, higher τ values are consistent with RT distributions with infrequent but overly prolonged RTs forming a larger rightward skew or tail of the corresponding distribution.

Leth-Steensen, Elbaz and Douglas [21] documented that higher variability in a simple reaction time task in cADHD is mainly attributable to heightened τ parameters, whereas μ and σ did not differ significantly when compared with normally developing controls. Using more complex inhibitory tasks (e.g. GoNogo), the RT distributions of cADHD were characterized by both larger τ and σ values, reflecting increased variability in the slow and fast portion of the distribution of RTs (see Figure 1) [19], [22], [23]. However, similar to simpler tasks, the data did not indicate a general slowing of responses indicated by non-significant differences in the parameter μ.

An examination of predictors of reaction times revealed that children with ADHD show a pronounced slowing of responses before and after omission errors [24]. These findings suggest that both omission errors and occasional prolonged RT indicated by the parameter τ may be linked to deficits in sustained attention. However, the exact nature of these “lapses of attention” and other possible explanations for increased IIV beyond attentional deficits are still being debated [17], [25]. Demonstrations of increased variability not only in the slow (τ) but also in the fast portion of the distribution (indicated by the parameter σ) suggest a potential multidimensional construct of IIV [26].

### 4. Summary and research questions

In a nutshell, the above mentioned findings suggest that a comprehensive understanding of inhibitory deficits in ADHD should take RT, both classical and distributional measures of IIV as well as commission and omission errors into account. To our knowledge, there is only one study which has analyzed IIV in inhibitory function with distributional measures in aADHD [27]. However, this study examined effects of smoking in a small sample without providing a healthy control group. Consequently it remains unclear, whether reduced stability of information processing accounts for a slowing of reaction times in inhibitory tasks in aADHD. Against this background, we aimed to examine response inhibition by applying a well-established GoNogo task in a sample of adult subjects with ADHD without medication and healthy controls matched for age, gender and education.

We hypothesize that slower RT in GoNogo tasks are due to an increased number of abnormally slow responses rather than a general slowing of performance. We therefore expect that in spite of a significantly prolonged mean RT in patients on the basis of classical RT measures, there will be no significant increase in the corresponding distributional parameter μ in aADHD in the present data. However, patients and controls will differ with regard to variability measures (SD) and especially with regard to abnormally slow responses measured by the parameter τ, which we assume to be significantly increased in patients. We further expect non-significant differences in commission errors between aADHD and controls. Together these hypotheses question a primacy of inhibitory deficits in aADHD.

Given findings that suggest a relationship between IIV (occasional slow responses) and sustained attention, we further expect a significant relationship between the parameter τ and the number of omission errors in aADHD. Given some evidence for a multidimensional construct of IIV in cADHD, we also examine whether differences between aADHD and controls are restricted to the parameter τ or also involve the parameter σ. Differences in the parameter σ would indicate that higher IV in aADHD cannot be exhaustively explained by occasional lapses of attention. Finally, we wanted to test the hypothesis if distributional parameters predict self-rated symptoms of inattention in aADHD.

## Methods

### 1. Subjects

The study sample consisted of a total of 80 adult subjects aged 19–65 years (M = 35.01 years; SD = 11.25 years) out of which 40 were diagnosed with ADHD and 40 were healthy control subjects matched for age, gender and education. All individuals participated voluntarily in the study and gave written informed consent prior to neuropsychological assessment. The study protocol was approved by the University of Heidelberg Ethics Review Committee and was conducted according to the Declaration of Helsinki.

The aADHD group consisted of outpatients recruited at the Department of Psychiatry and Psychotherapy – SRH Clinic Karlsbad-Langensteinbach (Germany). Diagnostic assessment was done by experienced clinicians including both a clinical psychiatric interview according to DSM-IV criteria [28] and the retrospective diagnosis of an ADHD in childhood (DSM-IV criteria). Childhood ADHD symptoms were self-rated with the short version of the Wender Utah Rating Scale (WURS-K; [29]. Severity of adulthood ADHD symptoms was self-rated with the ADHD self-report scale corresponding to the diagnostic criteria of DSM-IV [1], [30]. Patients were carefully screened and excluded (I) if they had clinically significant chronic medical conditions, (II) if they were currently treated with psychostimulants, (III) if there was a history suggestive of “psychosis” (indicating schizophrenia, delusional disorder, depressive disorder with psychotic features or bipolar disorder), (IV) if there was a history of neurological disorder including head injury, (V) if there was a history of substance abuse two months prior to the study, (VI) if the initial psychiatric assessment indicated a current major depressive episode or (VII) if the estimated verbal IQ was below 85. Within the aADHD group, N = 23 patients met DSM-IV criteria for ADHD combined type (aADHD-C) and N = 17 patients met criteria for ADHD predominantly inattentive type (aADHD-I). For 18 out of 40 patients with ADHD, there was anamnestic evidence for comorbid disorders, including minor to mild mood disorders (N = 14), anxiety disorders (N = 3), personality disorders (N = 4), eating disorders (N = 1) and substance abuse disorder (N = 1). At the day of the assessment 13 patients were being treated with antidepressive medication.

Data of 40 healthy control subjects were taken from the SCHUHFRIED database (COGBAT reference sample; N = 311; Schuhfried GmbH; Mödling, Austria) and matched to the aADHD group according to sex, education and age. In an unpublished questionnaire constructed by SCHUHFRIED none of the healthy control subjects reported to have a history of neurological and/or psychiatric disease or to take any medication or other substance known to affect the central nervous system. Moreover all controls filled out the Beck depression inventory (BDI II; [31]) and the Beck anxiety inventory (BAI; [32]) and were excluded in case of deviances from normality. Finally controls were excluded when there was evidence that the instruction of the test was not understood properly (e.g. strongly deviant response patterns in the GoNogo task). aADHD-Patients and healthy controls did not differ with respect to age (t(78) = −0.11, p = .820), gender (exactly the same distribution in both groups) or education (χ^2^(1) = 0.057; p = .811). Further information concerning age, sex and education is given in Table 1 for all groups.

**Table 1:** Distribution of subjects with regard to age, gender and education.

	adult ADHD (aADHD)	healthy Controls
N	40	40
**Age (years)** Mean ± SD	34.88±11.25	35.15±11.12
**Gender** (♂, ♀)	25, 15	25, 15
**Education** ^*^ Median, Range	3, 1–5	3, 1–5

**NOTE:** ^*^ Education was ordinally measured with 1 =  Compulsory schooling not completed (less than 9 years of school) or special school; 2 =  Completed compulsory schooling (9–10 years of school); 3 =  Completed vocational training (10–12 years of school); 4 =  Highschool graduation with university entrance exam (12–13 years of school); 5 =  University or college degree.

**Figure 2:**
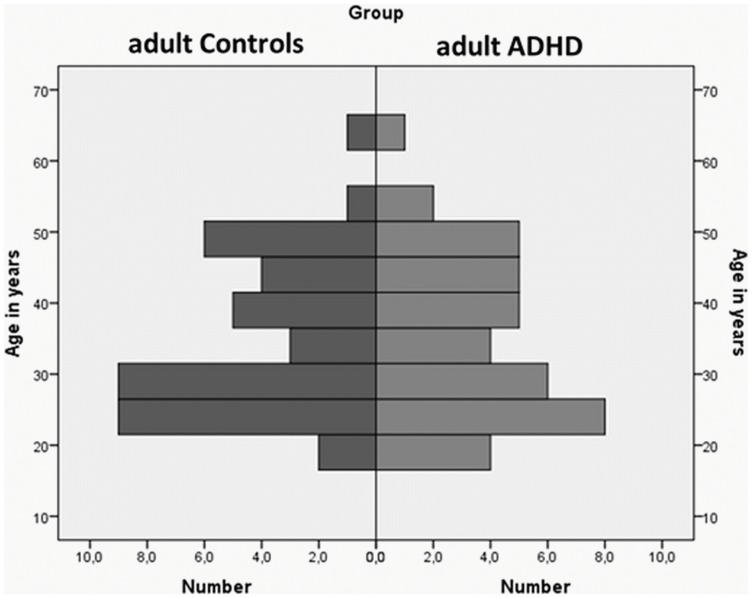
Distribution of age for both adult controls and adult ADHD.

Given the large age range in our sample, the corresponding distribution is illustrated in Figure 2.

### 2. Procedure

In order to assess response inhibition, the subtest GoNogo which is a part of the INHIB test of the Vienna Test System (VTS) was used. Subjects were seated in front of a computer screen and were required to press a button with their dominant finger when shown a triangle and refrain from pressing when shown a circle. After completing a block of practice trials, all subjects were instructed to work as fast and correct as possible. The stimulus requiring response (triangle) occurred in 80% of the trials ( =  Go Trials). Consequently the associated prepotent response had to be inhibited in 20% of the trials ( =  Nogo Trials) when a circle was shown. As the triangle is presented more often, the task creates a prepotency towards responding. Responding to circles results in commission errors, whereas not responding to a triangle leads to omission errors.

Each stimulus was presented centrally once every 1000 ms and remained on the screen for 200 ms. The sequence of trials was pseudo randomized. The task started with a first run consisting of 114 Go and 24 Nogo stimuli. After a short break allowing participants to rest, another 114 Go and 24 Nogo stimuli were presented. Overall, the completion of the task took about 20 min. The test's reliability (split-half reliability coefficients) is >.83 for the main variables [7].

### 3. Data Analysis

For data analysis all Go trials with reaction times less than 100 ms were discarded as “anticipatory” errors. For classical measures, primary variables of interest were mean reaction time (mRT), standard deviation of mean reaction time (SD) as well as the number of commission and omission errors.

Ex-Gaussian analyses were performed with the MATLAB toolbox “DISTRIB” according to Lacouture and Cousineau [33]. Data preprocessing and export to MATLAB was done with an individually tailored Excel macro. Estimates of the three ex-Gaussian parameters μ, σ and τ were obtained by fitting an ex-Gaussian distribution to the frequency distribution of correct Go responses for each subject. The fitting was done by an iterative search based on maximum likelihood criteria using the function “egfit.m”. The function “eglike.m” was used to return log likelihood values for all parameters. Finally, histograms with overlay ex-Gaussian probability functions were plotted with “plotegfit.m”. The number of RT observations used for each ex-Gaussian fit depended on the accuracy of responding. With M = 195 (SD = 13) and a range of 117 to 202 observations per subject, we were able to calculate sufficiently stable estimates for all parameters.

Statistical analyses were performed using SPSS 21 for Windows. Given violations of the normal distribution and the homogeneity of variances in various classical (see Figures S1–S2), ex-Gaussian (see Figures S5–S6) and error measures (see Figures S3–S4), we decided to use a nonparametric approach in order to obtain stable statistical analysis.

Consequently, we applied non-parametric U-tests in order to compare aADHD and matched controls with regard to classical (RT, SD), distributional (μ, σ, τ) and error measures. On the basis of the Holm-Procedure, these directional a priori hypotheses were adjusted according to an overall level of significance of α = .05. Additionally, effect sizes (d) were calculated for all comparisons in order to evaluate the magnitude of differences independent of sample size. For pairwise comparisons negligible effects (d>0.20), small effects (d = 0.20), medium effects (d = 0.50) and large effects (d = 0.80) were distinguished.

We computed non-parametric rank correlation coefficients (Spearman's Ρ) to test the relation between distributional measures and the number of omission and commission errors. Finally, we used non-parametric rank correlation coefficients in order to examine if distributional parameters are significantly correlated with the severity of self-rated ADHD symptoms in patients.

Moreover, given some unexpected findings, additional exploratory analysis was conducted in order to elucidate possible effects of comorbid depression on aADHD performance in the GoNogo task as well as possible differential effects of practice and/or fatigue for aADHD and controls in the course of our task. As there were no adequate non-parametric approaches to these questions, repeated measures (M)ANOVA was used.

## Results

The differences between aADHD-C and aADHD-I subgroups in both classical and distribution measures were non-significant (all p>.43); therefore, data from both groups was pooled for further statistical analysis.

### 1. Classical Measures (M, SD, Errors)

For the classical measures, patients with aADHD showed significantly slower reaction times (z = 3.43 p<.001; d = 0.76) compared with controls. Moreover, reaction times were more variable for aADHD as indicated by an increased SD (z = 3.38, p<.001; d = 0.88).

Though the number of errors of commission did not differ significantly between groups (z = 1.36, p = .172; d = 0.27), we found a significantly increased rate of omission errors in aADHD (z = 2.39, p = .017; d = 0.6). It is worth mentioning that two aADHD patients showed a largely increased number of omission errors. However, an exclusion of these patients did neither change group differences with regard to omission errors nor correlations of omission errors with other variables.

Based on a repeated measures MANOVA, exploratory analysis of time on task was used to exclude possible differential effects of practice or fatigue in aADHD and controls for RT, SD and errors. Non-significant results provided negative evidence for differential courses of reaction times, stability or accuracy in the first and second half of our GoNogo task in both groups (interaction effect group x time: F(76) = 0.64, p = .633).

### 2. Ex Gaussian modeling of mean reaction time distribution

**Figure 3:**
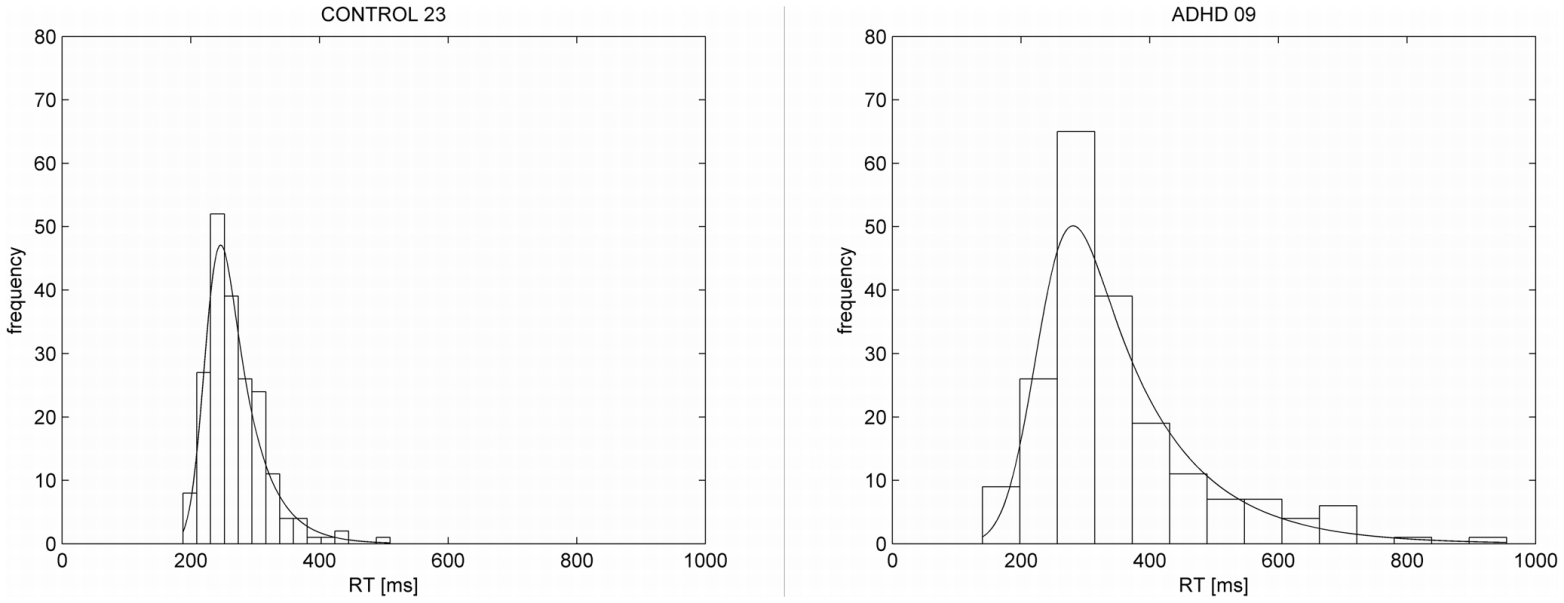
Frequency of intraindividual RTs with fitted ex-Gaussian probability functions exemplary for a control (left) and an ADHD (right) subject. NOTE: Please keep in mind that RTs in ADHD encompass a broader range (200<RT<1000 ms) compared with controls (200<RT<500 ms) resulting in broader frequency bins for the ADHD subject. Both subjects are comparable with regard to relevant demographic variables.

Ex-Gaussian parameters were estimated for every single subject. A comparison of chi-square fitting statistics indicated a comparable fit to RT data for both subjects with aADHD and controls (z = 1.77, p = .077) allowing further between group comparisons of distributional parameters. The parameter μ did not differ significantly between groups (z = 1.81, p = .070, d = 0.4). However, we found significant differences for the parameters σ (z = 2.43, p = .015; d = 0.54) and τ (z = 2.48, p = .013; d = 0.68) indicating higher variability around the mean and a higher number of occasional slow reaction times in aADHD (see Figure 3 below).

### 3. Correlational Analysis

Correlational analyses indicated a significant relationship between the parameter τ and the number of omission errors in the whole group (r = .57; p<.001; N = 80) as well as separately for aADHD (r = .67; p<.001; N = 40) and controls (r = .31; p = .049; N = 40). Moreover, we found significant correlations between μ and commission errors again for all subjects (r = −.61; p<.001; N = 80) as well as separately for aADHD (r = −.65; p<.001; N = 40) and controls (r = −.70; p<.001; N = 40).

However there were no significant correlations between distributional parameters μ, σ and τ with self-ratings of self-perceived ADHD symptoms in the aADHD group (r<.26; p>.113; N = 39). Furthermore, an additional exploratory analysis did not show any significant relationship between classical measures and symptom ratings (r<.28; p>.081; N = 39). As one ADHD patient did not return the questionnaires completely, correlational analyses were done with N = 39 instead of N = 40 patients.

## Discussion

Using a well-established inhibitory GoNogo task, we found robust differences in IIV in a sample of adult subjects with ADHD without medication when compared with healthy controls matched for age, gender and education. Controlling for IIV we did not find a significant slowing of performance in aADHD. In combination with our analysis of errors and their relationship to ex-Gaussian measures, the present data predominantly provide evidence for attentional rather than inhibitory dysfunction in aADHD, though increased IIV in aADHD may go beyond deficits in sustained attention. Distributional measures did not allow a prediction of self-rated attentional problems in aADHD.

### 1. Inhibitory function not significantly impaired in aADHD

One of the most prominent models of ADHD focuses on deficient inhibitory control as the core deficit of ADHD [4]. However, we found non-significant differences in errors of commission in aADHD compared to healthy controls. This finding speaks against an overt deficit in inhibitory control in aADHD. This is in line with the majority of studies employing GoNogo tasks in aADHD [8], [10], [11], [13] though Fisher, Aharon-Peretz and Pratt [34] documented an increased number of commission errors in a rather small sample using an auditory GoNogo task.

Applying the stop signal task, Boonstra, Kooij, Oosterlaan, Sereant and Buitelar [35] also suggested primary inhibitory dysfunction on the basis of significantly longer Stop Signal RTs in aADHD even after controlling for IQ and non-executive functions. This is in line with meta-analytic findings by Lijffijt, Kenemans, Verbaten and van Engeland [5] suggesting that inhibitory dysfunction in ADHD may even be more pronounced in adult subjects compared with children and adolescents. However, the vast majority of studies included in this meta-analysis – as well as – did not account for increased IIV of RT in aADHD, which may offer an alternative explanation to these “inhibitory deficits”. However, it is important to note that Stop Signal and GoNogo paradigms differ slightly from each other in terms of task demands and the exact cognitive processes involved, complicating direct comparisons of the Stop Signal literature with the current task. While the Stop Signal task requires the inhibition of an already initiated response and consequently focusses on the motoric component of inhibition, the GoNogo task additionally requires a decision whether a stimulus needs an inhibition or not [7].

Though longer RT in classical measures in aADHD in the present GoNogo data suggest a generally decelerated performance in patients, non-significant differences in the distributional parameter μ in aADHD and controls speak against a significant slowing of inhibitory function in aADHD. Given significantly higher IIV in aADHD as indicated by values of the distributional parameter τ, we argue that slower mean RT in patients are not simply due to a generalized slowing of performance but rather due to an increased proportion of occasional slow responses. Though corresponding distributional results are lacking in aADHD, our findings are largely in line with ex-Gaussian results observed in children and adolescents with ADHD [19], [23], [36].

However, though non-significant, the present data reveal a trend towards larger μ values in aADHD compared to healthy controls. In an additional analysis we were able to rule out a possible influence of former depressive symptoms in subjects with aADHD, which may have persisted and reduced the speed of information processing relative to healthy controls. Furthermore, comparisons of mean RT in the first and second half of our task document a similar course of RT in both groups speaking against differential effects of motivation or fatigue in aADHD and controls. Given a strong negative association between the number of commission errors and the parameter μ in aADHD in the present data, we cannot rule out that a borderline slowing in patients may represent some compensation of subtle inhibitory deficits. Future studies, which additionally use a diffusion model framework allowing to measure speed-accuracy trade-offs more directly, may help to better understand possible compensatory mechanisms in aADHD in GoNogo tasks [25].

Summing up, we found evidence for minor inhibitory dysfunction in aADHD. This is hardly compatible with models, which suggest inhibitory dysfunction to be the core deficit of ADHD. Against the background of former results observed in cADHD indicating faster and possibly more impulsive responses [19], [25], the present data are compatible with a decrease of at least overt inhibitory dysfunction with age. These changes may be paralleled by a reduction of symptoms of hyperactivity and impulsivity during adolescence [9], [37]. Halperin, Trampush, Miller, Marks and Newcorn [38] have provided preliminary cross-sectional support for the hypothesis that recovery from ADHD over the course of development may be associated with improvements in executive control functions whereas more basic cognitive deficits persist and are not related to symptom status. However, there is recent longitudinal evidence from Coghill, Hayward, Rhodes, Grimmer and Matthews [39] suggesting that improvements in executive functions do not explain clinical improvements. This is in line with recent meta-analytic findings [40] who found no evidence that more consciously controlled neurocognitive functions differentiated ADHD persistence from remittance in young adults. Beyond the heterogeneity of neuropsychological profiles in ADHD, the use of different measures for executive functions may account for these deviant results. In this context, it is worth mentioning that hardly any of the above mentioned studies included GoNogo tasks or distributional measures of IIV in order to measure inhibitory function.

### 2. Clear evidence for attentional dysfunction in aADHD

We found robust indications for attentional problems in aADHD [41] as reflected in increased IIV [17] and a significantly larger number of errors of omission in patients as compared to controls. The latter finding is in line with other studies documenting increased numbers of omission errors in aADHD in inhibitory [8] and attentional tests [37]. Moreover, we found a strong positive correlation between the number of omission errors and IIV (as given by the number of abnormally slow responses: τ) in both aADHD and control subjects. Given this result, we speculate that failures of sustained attention, which did not last long enough to produce errors of omission, may have resulted in an abnormally prolonged RT producing higher variability in the slow portion of the distribution of RTs in aADHD. This assumption is in line with a study of Epstein, Hwang, Antonini, Langberg, Altaye, et al. [24], who revealed that children with ADHD show a pronounced slowing of responses before and after omission errors. Cheyne, Solman, Carriere and Smilek [42] supposed that “attentional lapses” may begin with transient disengagement of attention, then move to automatic responding without actively attending ( =  prolonged RT, indicated by τ) and finally result in “mind wandering”, which produces omission errors.

Regarding the neural basis for attentional lapses, there is evidence that occasional slow responses are paralleled by failures to suppress activity in the default mode network (DMN) [43]. As a counterpoint to brain areas involved in attentional control, the DMN supports internally directed mental activity [44]. Consequently activations of the DMN during a task may indicate a shift away from goal directed behaviour or mind wandering. In this context it is worth mentioning that children with ADHD show problems deactivating the DMN which were indeed related to IIV [45]. Applying a Diffusion model and ex-Gaussian measures to children with ADHD, Karalunas and Huang-Pollock [25] were able to show that slower and less efficient information processing in cADHD predicts deficits in inhibitory control measured by a Stop Signal task. Interestingly these deficits in information processing were correlated with the parameter τ, suggesting that there is a link between speed and efficiency on the one and the number of slow responses in cADHD on the other hand. However, according to Matzke and Wagenmakers [20] such comparisons should be done with caution as ex-Gaussian parameters do not correspond uniquely to parameters of the diffusion model.

Though increased numbers of omission errors and prolonged RTs in aADHD as found in the present study may – among other things – reflect occasional lapses of attention, the present data additionally revealed that higher IIV as indicated by classical measures of SD is both due to the above mentioned increase of abnormally slow responses ( =  τ) and an increased variability of RT around the mean ( =  σ). In other words, the present findings suggest that aADHD is characterized by generally increased variability affecting both the slow and the fast portion of the RT distribution (see Figure 1 or Figure 3 for illustration). Our findings correspond well to aADHD data published by Epstein, Hwang, Antonini, Langberg, Altaye, et al. [24], who – using an attentionally demanding task – were not able to fully explain increases in IIV of patients with aADHD by attentional lapses. In deviation from the present findings, a recent meta-analysis by [17] concluded that ADHD-related variability appears to be primarily attributable to a subset of abnormally slow responses ( =  τ), rather than ubiquitous variability across all trials in a given task ( =  σ). However, studies which employed complex tasks (requiring attentional or inhibitory control) in aADHD were under-represented in this meta-analysis. This is of special interest, as – with regard to cADHD – there is some evidence that while simple RT tasks merely go along with an increase of τ [21], [46], performance in more complex tasks requiring attentional or inhibitory control is characterized by an increase in both τ and σ [23], [47]. The present findings provide first evidence that – similar to cADHD – increased IIV in aADHD may be the sum of both a subset of abnormally slow responses and a more general increase of variability in all responses. This is in line with a multidimensional construct of IIV [26].

While increases in the number of slow responses (or the parameter τ), are more or less explicitly integrated in “lapses of attention” models [21], heightened σ values probably go beyond corresponding deficits in sustained attention. In cADHD, an increase in σ was proposed to reflect fundamental inefficiency in mechanisms critical to engage a state of preparedness to respond [23]. In the present data, non-significant relationships between σ and both omission and commission errors do not suggest a relation to dysfunction of sustained attention or inhibition. Again future studies applying Diffusion model frameworks to attentional and executive tasks may prove helpful in order to disentangle some of multiple interacting processes like stimulus encoding, information processing, motor preparation and speed-accuracy trade-off effects [25]. To date neuroimaging research has linked IIV to both abnormal frontal lobe grey and white matter anatomy as well as abnormal functional activation of frontal areas and the DMN. In line with frontal lobe abnormalities some lesion studies suggest that IIV in performance may be a more general index of the efficiency with which executive control processes are implemented [48], [49]. However, as these findings are based on classical measures of IIV, it remains unclear whether this relationship is due to a pattern of infrequent slow responses, an increased variability in all responses or both. Summing up, we suggest that increased IIV in aADHD affects both the slow and the fast proportion of the RT distribution and cannot be fully explained by “attentional lapse” models of ADHD.

Integrating both results on inhibitory and attentional function, we conclude that our data are not compatible with a primary deficit of inhibition in aADHD. This conclusion applies to both patients of the combined (ADHD-C) and the predominantly inattentive type (ADHD-I). In fact, our findings highlight attentional dysfunction in aADHD. Performance was not so much characterized by a general slowing, which may indicate compensatory processes due to inhibitory dysfunction but by an increased number of abnormally slow responses and associated omission errors. However, heightened IIV in aADHD was not restricted to these higher numbers of slow responses but affects both the slow and the fast portion of the RT distribution tentatively suggesting executive dysfunction beyond attentional lapses [26].

### 3. Implications for neuropsychological testing

Based on the present findings, we suggest that omission but not commission errors – in combination with measures of IIV – may be an adequate variable for identifying cognitive deficits in inhibitory function in aADHD within neuropsychological assessment. However, given the large heterogeneity of cognitive profiles in aADHD, diagnostic conclusions on the basis of single cognitive functions are not acceptable. It is perfectly possible that the heterogeneity in ADHD is due to meaningful subgroups beyond the combined and predominantly inattentive type, which should be the target of future studies.

We used distributional analysis to elucidate basic principles of neuropsychological dysfunction in aADHD which could not be differentiated on the basis of classical measures of RT and corresponding SD. However, it is interesting to note that effect sizes for variability measures based on a distributional (σ: d = 0.54; τ: d = 0.68) approach are not larger when compared with a classical (SD: d = 0.88) approach. Therefore, – taking a solely diagnostic point of view – we tentatively suggest that classical measures are as useful in differentiating aADHD subjects from normal controls as more costly/complex distributional ones. However, this should be investigated in future studies using logistic regressions and/or receiver operating characteristic curves.

### 4. Limitations and future directions

Given the exploratory nature of some analysis and the large age range included in our data, the results from the current study should be interpreted with caution and call for replication by independent data. However, with regard to the large age range in the current study, it should be noted that an explorative ANCOVA with the covariate age did not produce a significant effect for the variable age. Moreover the pattern of significant results in neuropsychological dependent variables was unchanged when compared with U-tests. As far as non-parametric testing in the current study is concerned, it is worth mentioning that the use of parametric t-tests instead of u-tests did not change the overall pattern of significant results at all.

Regarding comorbidity, a possible impact of depressive symptoms and linked antidepressant medication in the current sample cannot be ruled out. Although we cannot completely rule out possible influences of depressive symptoms/effects of antidepressant medication, it should be noted that we did not find significant differences between aADHD patients with and without depressive symptoms and a corresponding medication in neuropsychological variables.

Although we found clear evidence that ADHD is characterized by increased IIV compared with healthy controls, seemingly similar abnormalities can be found in autism [50], schizophrenia and depression [51], traumatic brain injury [52] and early stage dementia [53]. Consequently, increased intraindividual variability may not be specific to ADHD but a more general marker for problems with attention, executive function and/or some kind of psychopathological process. However, most of the above mentioned studies used classical measures in order to compute IIV. Using distributional measures, Rentrop, Rodewald, Roth, Simon, Walther, et al. [54] found increased values of τ but not σ in a sample of high functioning patients with schizophrenia. Unfortunately we did not include different clinical conditions (including schizophrenia, depression and possibly neurologic disorders) allowing us to examine possible differences in IIV in more detail. In addition to distributional analysis, it might also be important to apply Diffusion models to aADHD data in order to disentangle multiple processes involved in speeded RT tasks in different disorders. Finally, future studies should extend the present approach to more comprehensive subsets of attentional and executive tasks in order to provide additional insight into cognitive mechanisms characterizing adult but also childhood ADHD. Such work has theoretical implications for etiological models of ADHD as well as more practical implications for neuropsychological testing in aADHD.

## Supporting Information

Figure S1 **Distribution of mean RT for both adult ADHD patients and healthy controls.**
(TIF)

Figure S2 **Distribution of the SD of mean RT for both adult ADHD patients and healthy controls.**
(TIF)

Figure S3 **Distribution of commission Errors for both adult ADHD patients and healthy controls.**
(TIF)

Figure S4 **Distribution of omission Errors for both adult ADHD patients and healthy controls.**
(TIF)

Figure S5 **Distribution of Mu (μ) both adult ADHD patients and healthy controls.**
(TIF)

Figure S6 **Distribution of Sigma (σ) for both adult ADHD patients and healthy controls.**
(TIF)

Figure S7 **Distribution of Tau (τ) for both adult ADHD patients and healthy controls.**
(TIF)
