# Supplementary figures and images for: Intraindividual Variability in Inhibitory Function in Adults with ADHD – An Ex-Gaussian Approach
Source: PLoS One. 2014 Dec 5;9(12):e112298. doi: 10.1371/journal.pone.0112298 (PMC4257533; doi:10.1371/journal.pone.0112298)

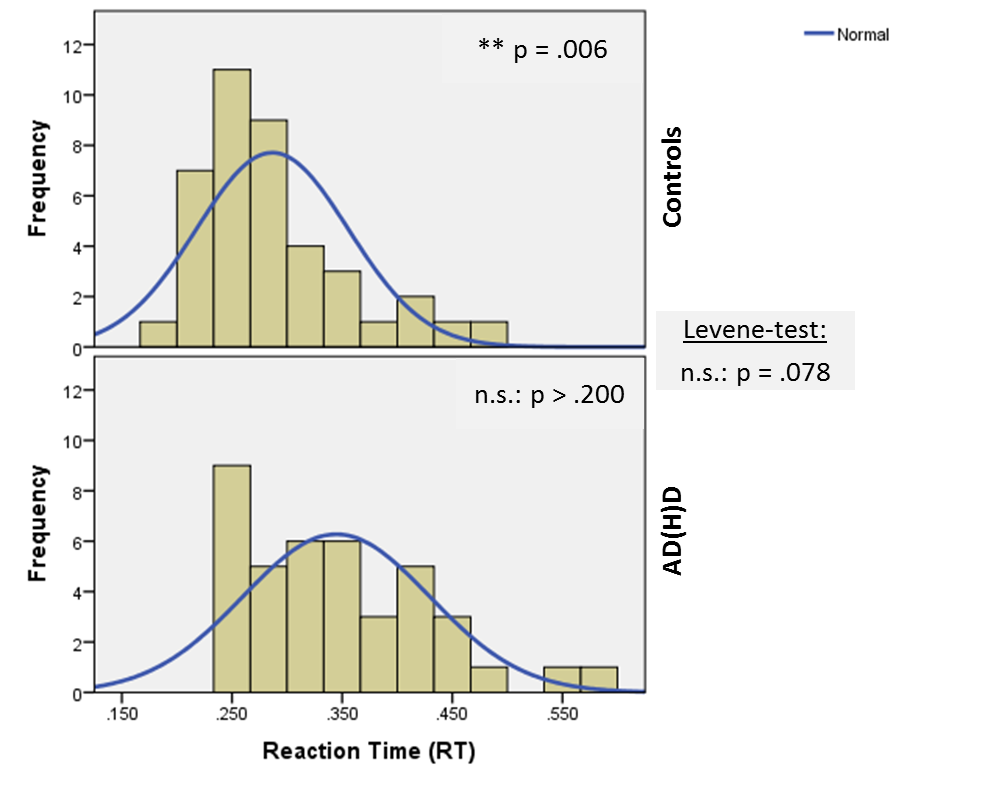

Supplement: Figure S1 — Distribution of mean RT for both adult ADHD patients and healthy controls. (TIF) [file pone.0112298.s001.tif]

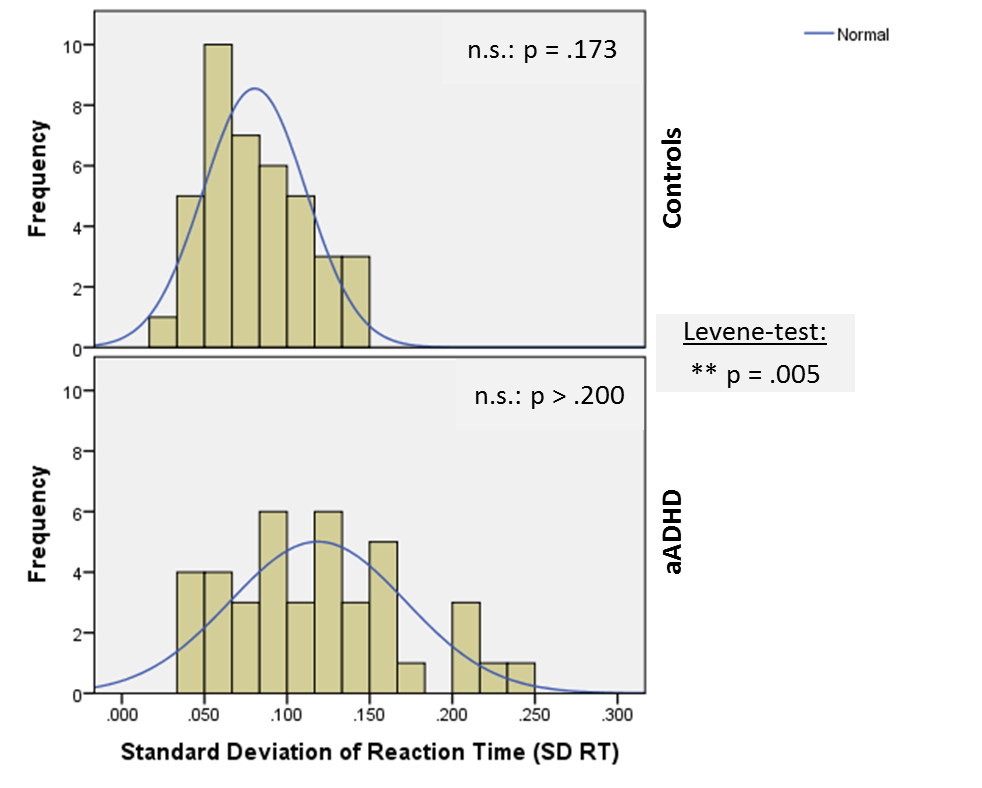

Supplement: Figure S2 — Distribution of the SD of mean RT for both adult ADHD patients and healthy controls. (TIF) [file pone.0112298.s002.tif]

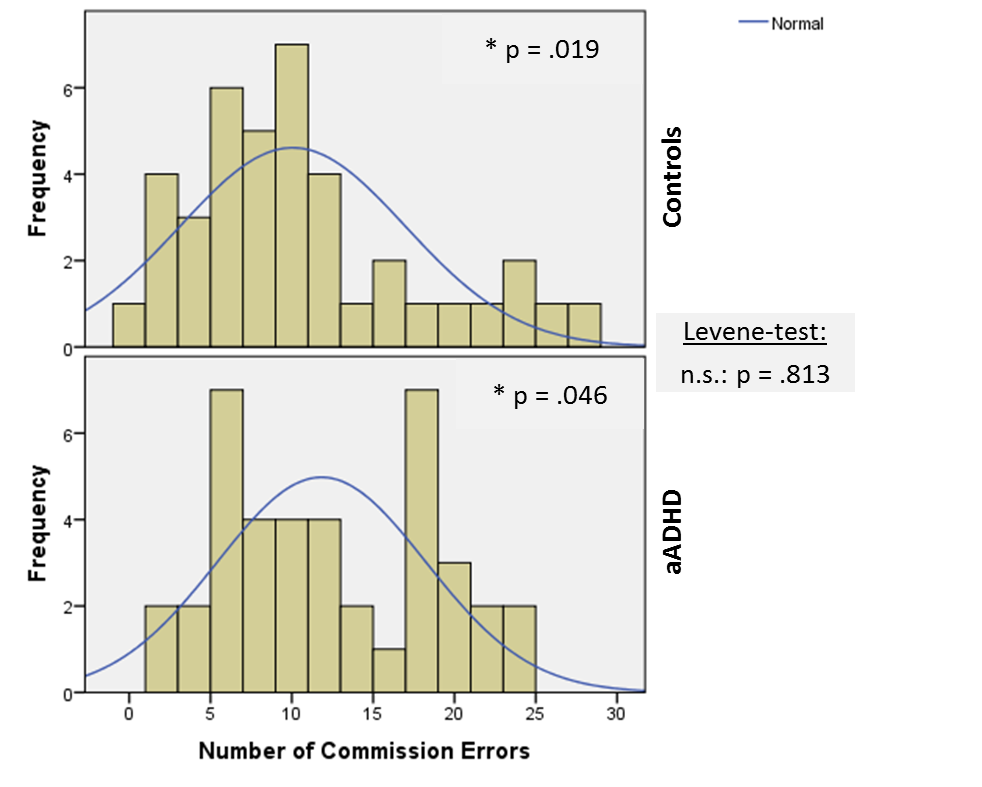

Supplement: Figure S3 — Distribution of commission Errors for both adult ADHD patients and healthy controls. (TIF) [file pone.0112298.s003.tif]

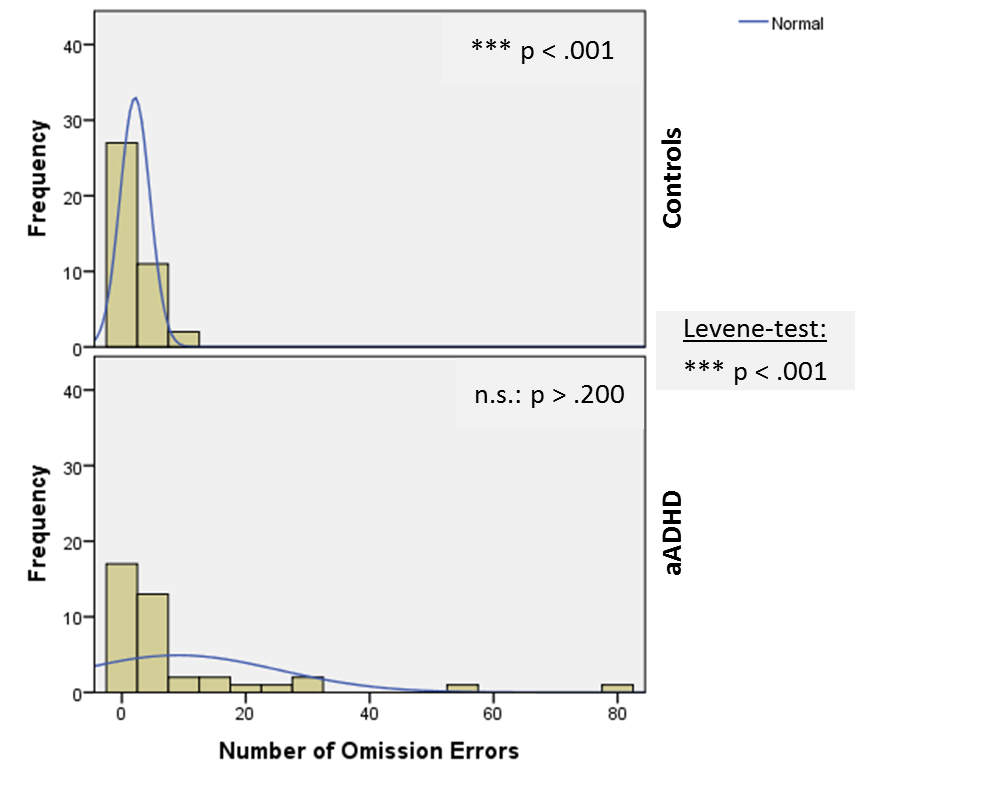

Supplement: Figure S4 — Distribution of omission Errors for both adult ADHD patients and healthy controls. (TIF) [file pone.0112298.s004.tif]

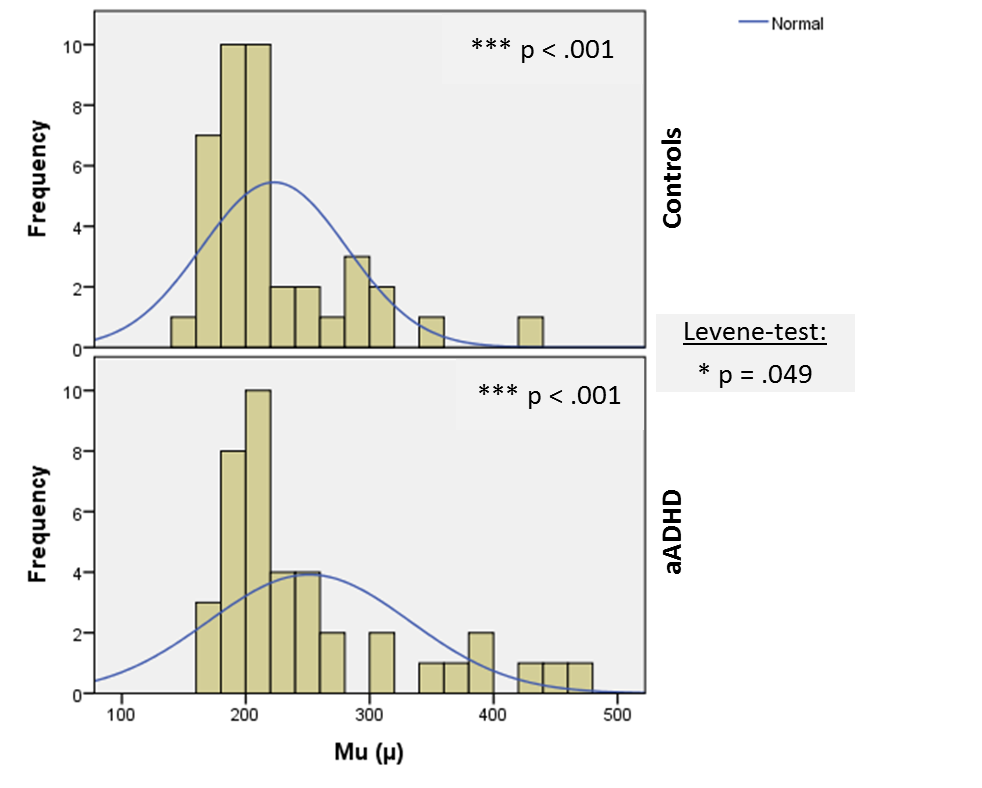

Supplement: Figure S5 — Distribution of Mu (μ) both adult ADHD patients and healthy controls. (TIF) [file pone.0112298.s005.tif]

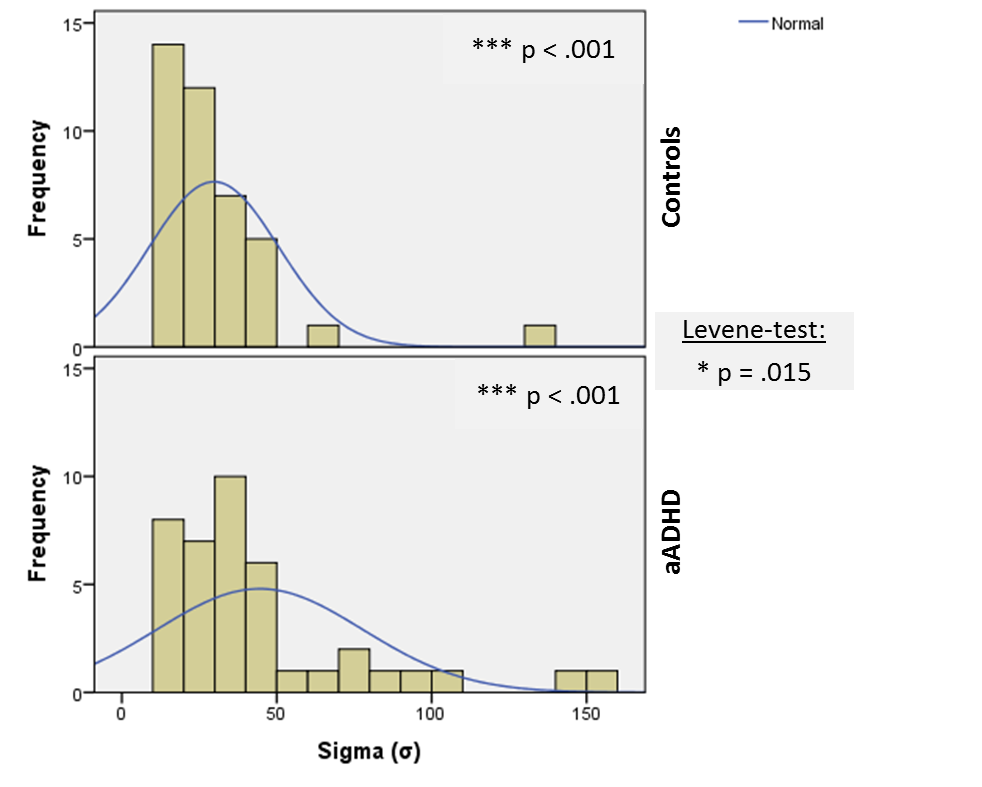

Supplement: Figure S6 — Distribution of Sigma (σ) for both adult ADHD patients and healthy controls. (TIF) [file pone.0112298.s006.tif]

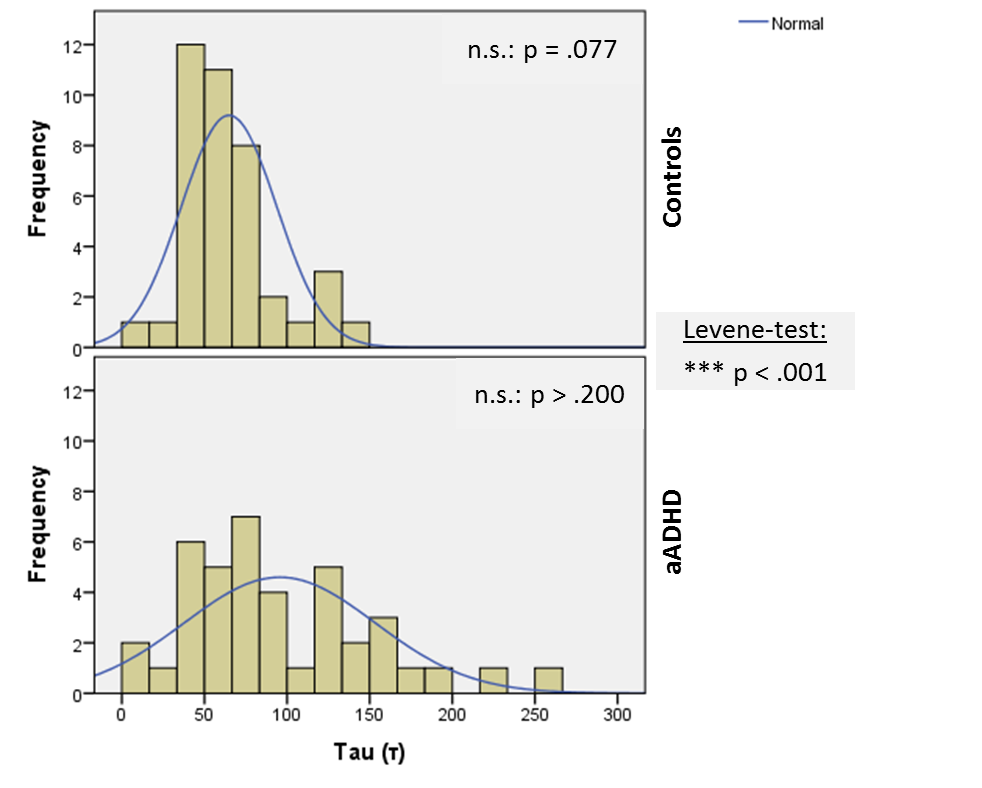

Supplement: Figure S7 — Distribution of Tau (τ) for both adult ADHD patients and healthy controls. (TIF) [file pone.0112298.s007.tif]
